# Anatomical and Neuromuscular Determinants of Strength Change in Previously Untrained Men Following Heavy Strength Training

**DOI:** 10.3389/fphys.2019.01001

**Published:** 2019-08-06

**Authors:** J. Trezise, A. J. Blazevich

**Affiliations:** School of Medical and Health Sciences, Centre for Exercise and Sports Science Research (CESSR), Edith Cowan University, Joondalup, WA, Australia

**Keywords:** strength training, linear models, cross-sectional area, fascicle angle, muscle activity

## Abstract

This study examined whether changes in strength following a moderate-duration strength training program were associated with changes in specific combinations of anatomical and neuromuscular variables. 36 men (18–40 y) completed 10 weeks of lower-limb heavy resistance (6-RM) strength training. Measurements included cross-sectional area (CSA), fascicle length (l_f_) and fascicle angle (θ_f_) from proximal, middle and distal regions of the four quadriceps components; agonist (EMG:M), antagonist (EMG) muscle activities and percent voluntary quadriceps activation (%VA; interpolated twitch technique); patellar tendon moment arm distance; and maximal isometric, concentric and eccentric (60° s^–1^) torque. Multiple regression models were developed to quantify the relationship between the change in maximum torque and the changes in combinations of anatomical and neuromuscular variables. The best model for each contraction mode was determined using Akaike’s Information Criterion (AIC_c_), an information-theoretic approach for model selection. Strength increased significantly following training (mean range = 12.5–17.2%), and moderate relationships were observed between modeled data (using best-fit prediction models) and the change in torque for each contraction mode. The change in isometric torque was best (although weakly) predicted by the linear combination of the change in proximal-region vastus lateralis (VL) CSA and fascicle angle (*R*^2^ = 0.27, *p* < 0.05; AIC_c_*w_i_* = 0.52, i.e., the probability the model would be selected as the “best model”). The models best predicting the change in concentric and eccentric torque both included the combination of the change in quadriceps (i.e., mean of all muscles) EMG:M and the change in vastus intermedius fascicle angle combined with either a change in proximal-region VL (*R*^2^ = 0.40, *p* < 0.001; AIC_c_*w_i_* = 0.15) or whole quadriceps (*R*^2^ = 0.41, *p* < 0.001; AIC_c_*w_i_* = 0.30) CSA (concentric and eccentric, respectively). Models incorporating the change in proximal CSA typically received substantial support (AIC_C_ < 2) for concentric torque prediction models, and the change in % VA and pre-training moment arm distance had substantial support for use in eccentric torque prediction models. In conclusion, adaptations varied between individuals, however strength training programs targeted to improve a group of variables that particularly includes agonist muscle activation might yield the greatest improvements in concentric and eccentric knee extension strength, whereas proximal muscle size and fascicle angle appear most important for isometric torque improvements.

## Introduction

Strength training, especially in previously untrained individuals, elicits substantial functional and structural adaptations leading to increases in muscular strength. However, these neuromuscular (Maughan et al., 1983; Higbie et al., 1996; Hubal et al., 2005; Erskine et al., 2010) and strength (Hubal et al., 2005; Blazevich et al., 2008; Erskine et al., 2010) adaptations vary markedly between individuals. Muscle size, for example, is considered an important factor influencing strength expression and can account for ∼60% of the inter-individual variability in strength in non-strength trained adults (Maughan et al., 1983; Fukunaga et al., 2001; Blazevich et al., 2009; Trezise et al., 2016) yet gains in muscle size have been found to be less strongly related to training-induced strength improvements (Jones and Rutherford, 1987; Erskine et al., 2010; Ahtiainen et al., 2016). Strength training also elicits adaptations in muscle architecture (Kawakami et al., 1995; Aagaard et al., 2001; Blazevich et al., 2007; Seynnes et al., 2007) and activation (Häkkinen et al., 1985a; Narici et al., 1989; Aagaard et al., 2000b; Del Balso and Cafarelli, 2007) so changes in these neuromuscular variables may confound the relationship between muscle size and strength, and potentially explain the larger inter-individual variability in strength improvements following training. Thus, the combined effects of clusters of variables may be more important to study than the influence of single variables. To date, however, this speculation has received relatively little scientific scrutiny so the relative importance of different neuromuscular variables (or clusters of neuromuscular variables) to the training-induced strength increase is not known. Additionally, with the large heterogeneity in individuals’ adaptations to training (Ahtiainen et al., 2016) the relationships between the changes in neuromuscular variables also need to be explored. Given this, and despite a wealth of research detailing the neuromuscular responses to training, specific neuromuscular targets have not been identified in which large changes might lead to the greatest improvement in muscular strength.

Knee extensor torque production in particular is required for the successful completion of many activities of daily living (e.g., locomotion, chair sitting and rising, stair climbing) and athletic tasks, so it is an important muscle group for study. In our previous study (Trezise et al., 2016), quadriceps muscle size and activation [both the amplitude of agonist muscle EMG activity and percent voluntary activation assessed using interpolated twitch technique (ITT)] and vastus lateralis fascicle angle were identified as the best predictors of maximum isometric and eccentric knee extension torque, while muscle size, fascicle angle and patella tendon moment arm distance collectively were the best predictors of maximal concentric torque. However, it is possible that the variables most predictive of maximum strength within a population (i.e., in a cross-sectional analysis) have a different impact on the strength changes elicited by a training intervention (i.e., in a longitudinal analysis) since time-dependent (within-participant) changes may be considerably less than the between-subject variation. Thus, longitudinal studies are required in order to identify the neuromuscular factors most associated with longer-term strength change. The identification of the neuromuscular variables that most influence strength change would allow for the targeting of these variables with specific exercise training regimes, and the provision of individualized training programs based on a person’s structural and/or functional characteristics.

Given the above, the present study was designed to determine whether changes in strength (isometric, concentric and eccentric) following moderate-duration (10 weeks) high-resistance (6-repetition maximum [6-RM]; ∼85–90% maximum load) strength training were associated with changes in specific, or clusters of, neuromuscular variables. Determination of the strongest relationships between changes in strength and changes in the anatomical and neuromuscular variables can provide insight as to the most relevant mechanisms influencing strength change. As anatomical and neuromuscular adaptations to strength training are known to be load, volume and velocity dependent (Häkkinen et al., 1985b; Blazevich et al., 2003; Bloomquist et al., 2013) it is important to emphasize that the present research explored the effects of heavy, and thus slow-speed, lower-limb strength training. Also, as many activities of daily living and athletic tasks require the performance of isometric and dynamic contractions, it was considered important to determine the relationships between the measures of anatomical structure and neuromuscular function versus isometric, concentric and eccentric strength. Finally, as the mechanisms influencing strength change likely alter as training progresses, we specifically chose to study adaptations in the first weeks of training (10 weeks) since the magnitude of strength change in previously untrained individuals in this period is likely to influence their likelihood of continuing the training in the longer term.

## Materials and Methods

### Participants and Experimental Protocol

Thirty-six healthy untrained men (29.0 ± 5.1 y; 1.78 ± 0.05 m; and 78.9 ± 8.2 kg) between the ages of 19 and 40 years volunteered to participate in, and subsequently completed, this study. Four additional volunteers who began the study were not included in the final analysis: two dropped out due to work commitments, and two were excluded based on personal circumstances obstructing their training during the last 2 weeks. The participants were classified as untrained based on their response to a metabolic work rate questionnaire (Ainsworth et al., 2000). All participants had an average weekly metabolic energy equivalent score (MET) of <30/day and had not performed any regular lower-limb strength training in the past 4 years. Participants were excluded if they suffered from cardiovascular or inflammatory disease, a lower-limb injury within the last 3 months, or any other condition that could affect performance during the testing and training protocols. Prior to participation, they were provided written informed consent. The experimental procedure was approved by the Institutes’ Human Research Ethics Committee and the study was conducted in accordance with the Declaration of Helsinki.

The participants performed six testing sessions across a 2-week period to measure muscle size and architecture, maximal isometric voluntary torque and muscle activation capacity, maximal voluntary isokinetic torque and activation capacity, and patellar tendon moment arm distance. Each session was separated by at least 48 h. They then attended one pre-training gym session to determine their maximum load for 6 repetitions of each exercise and to familiarize themselves with the training exercises. Post-training testing began 4–5 days after their final training session to allow recovery of strength and for fluid shifts to stabilize, and all testing was then completed over a 5-day period. Each participant completed all test sessions at the same time of day (± 2 h) (Pearson and Onambele, 2006).

### Training Program

The participants completed two training sessions per week for 10 weeks (20 sessions). All training sessions were supervised and the participants were required to complete at least 18 training sessions. The exercise protocol consisted of incline (45°) leg press, knee extension and leg curl exercises against a heavy load on commercial fitness machines (Cybex International Inc., Medway, MA, United States). The participants performed 3 sets of 6 repetitions per exercise (6-RM). The load was progressively increased across the 10 weeks. During each set, if the participant managed 6 repetitions, depending on the ease with which the set was completed, the load was either maintained or increased by ∼5% for the subsequent set. If the participant managed only 5 repetitions, then they were assisted (spotted) to reach the 6^*th*^ repetition and the load was decreased ∼5% for the next set. These loads and volumes were chosen as they have previously been shown to stimulate substantial strength and hypertrophic adaptations (Kraemer and Ratamess, 2004). The first session was completed at 60% 6-RM to both accustom the participants to the training exercises and minimize muscle soreness, and all subsequent sessions were completed at 100% 6-RM. Two minutes of passive rest was given between sets and 3 min was allowed between exercises; rest periods were strictly enforced by the training supervisor. To control the range of motion, participants were required to cover the range of 10–90° for knee extension, and 5–90° for the leg press. This was controlled by the use of individualized markers taped to the exercise equipment for the participants to aim for. The participants were verbally encouraged throughout each session to give their maximal effort. The warm-up for each session consisted of 5 min of low-intensity, self-paced stationary cycling and 2 warm-up sets of 6 repetitions of each exercise at approximately 50 and 70% of the day’s load. The warm-down consisted of 5 min of cycling and 5 min of static stretching. As post-exercise ingestion of protein assists in eliciting an optimum training response (Phillips, 2004) and individual variations in post-exercise nutrition might increase training adaptation variation, all participants consumed a protein shake immediately post-training (Redbak Whey Protein, International Health Investments Pty Ltd., Helensvale Queensland). This contained between 20 and 40 g (0.4 g protein per kg body mass) of whey protein isolate powder comprised of 86% protein and 8% of both carbohydrates and fats. Participants were also instructed as to the need to have an adequate energy intake (including proteins, carbohydrates and fats) during the 10-week training period.

### Testing Procedures

The testing protocol has been explained in detail elsewhere (Trezise et al., 2016) but will be briefly described below.

### Isometric Torque and Neuromuscular Measurements

To measure maximal voluntary and electrically elicited isometric knee extension torques the participants performed maximal voluntary isometric contractions (MVCs) whilst sitting in a custom-built isometric dynamometer. As maximum isometric torque varies with joint angle, the angle of maximum torque production was found for each individual by performing MVCs at a range of 5–7 sequential angles with 5° increments (0° = full knee extension). Based on an estimated location of each participant’s peak torque angle from their familiarization sessions, participants began their MVC efforts at either 45°, 55° or 65°. To avoid the greater fatigue associated with performing a maximal efforts at longer muscle lengths (Desbrosses et al., 2006), the contractions progressed from an extended (i.e., short muscle length) to a flexed (i.e., long muscle length) position. The maximum isometric torque for each participant was taken as the maximum torque (T_ISO_) at any angle during the 500 ms prior to the superimposed twitch (explained below).

Each MVC was held for 3 s and a single (Behm et al., 1996) supramaximal electrical stimulus (140% M_max_ current intensity) was applied to the femoral nerve 2 s before contraction onset, during the torque plateau and 2-s after each MVC (see electrical stimulation protocol below). Two MVCs were performed at each angle, but if the peak torque values differed by >5 N⋅m a third MVC was completed. Participants had a 1-min rest between MVCs at the same angle, and 2-min rest between joint angles. To ensure the MVCs were not influenced by fatigue, the first joint angle was retested to confirm that fatigue was not induced throughout the testing (identified as >5% decrease in MVC from the initial trial). Strong verbal encouragement was provided during each MVC and a computer screen displaying real-time visual feedback of the torque data was displayed in front of the participants. The greatest torque at each angle was used for T_ISO_ analysis, while the peak unpotentiated (i.e., pre-MVC) twitch torque (T_Un–TW_) and peak potentiated (i.e., post-MVC) twitch torque (T_Pot–TW_) variables were defined as the mean obtained during the two strongest contractions. Intra-session reliability testing of the electrically induced and voluntary torques for eight participants produced coefficients of variation [CV (mean ± SD)] of 1.2 ± 0.9%, 3.6 ± 1.2% and 1.5 ± 1.4%, for T_ISO_, T_Un–TW_ and T_Pot–TW_, respectively.

### Electrical Stimulation Protocol

Percent voluntary activation, unpotentiated and potentiated muscle twitch torques, and the maximum muscle compound action potential (M-wave) amplitude were determined using a single supramaximal (140% M_max_ current intensity) electrical stimulus to the femoral nerve. This intensity ranged from 98 to 560 mA across the participants, with the majority between 180 to 280 mA. The single 2-ms (400 V) rectangular pulses were delivered via a high-voltage constant-current stimulator (Digitimer, model DS7AH, Welwyn Garden City, United Kingdom). The femoral nerve was located via ultrasonography while the participant sat in the custom built chair with a knee joint angle of 70°. The cathode was placed 0.5 cm medial and inferior to the femoral nerve, and the anode 2 cm lateral and superior to this position; to produce the greatest M-wave response at submaximal intensity the cathode position was altered slightly if required.

### Concentric and Eccentric Torque Measurements

Maximum concentric and eccentric knee extension contractions were performed at an angular velocity of 60°⋅s^–1^ on an isokinetic dynamometer (Biodex System 3, Biodex Medical Systems, Shirley, New York, NY, United States). This angular velocity provided a 1.5-s concentric phase, which was similar to that used during training exercises and ensured that slow-speed, high-force muscle strength was tested. Range of motion (ROM) for both concentric and eccentric contractions was 100° to 15° (0° = full extension), and torque signals were corrected for gravity. Following a warm up, one set of three repetitions of concentric and eccentric knee extensor contractions were completed. A second set was completed if the two peak torque values differed by >5 N⋅m. A 3-s rest was imposed between repetitions (30°⋅s^–1^ lever arm return speed) and a 2-min rest was allowed between sets. The maximum torque values (T_CON_ and T_ECC_) were used for analysis. Strong verbal encouragement was provided during each contraction and a computer screen displaying real-time visual feedback of the torque data was displayed in front of the participants. Intra-day and inter-day reliability testing (1-wk interval) for the three maximal concentric and eccentric knee extension contractions in 10 participants yielded CVs of 2.5 and 3.4% (intra-day) and 4.1 and 1.4% (inter-day), respectively.

### Muscle Activation

Electromyogram signals (EMG) were obtained using bipolar silver/silver chloride surface electrodes (10 mm diameter; Kendall Healthcare, Medi-Trace^TM^ 200 Series, United States) from rectus femoris (RF), vastus lateralis (VL) and vastus medialis (VM) and the long head of biceps femoris (BF) during the isometric and isokinetic contractions. The electrodes were positioned according to SENIAM guidelines (Hermens et al., 2000), and the reference electrode was placed on the proximal shaft of the tibia. The EMG signals were collected at an analog-digital conversion rate of 1 kHz and filtered using a fourth-order, zero-lag band pass (10–500 Hz) Butterworth filter.

Maximal isometric EMG was measured as the root mean square (RMS) 500 ms before superimposed stimulation (Maffiuletti and Lepers, 2003; Millet et al., 2003). Isokinetic RMS EMG was measured over a 30° range (covering 500 ms) around the peak torque angle. All agonist muscle contractions were then normalized to the unpotentiated M-wave amplitude (EMG:M-wave ratio). For both the isometric and concentric MVCs, antagonist EMG was normalized to maximal EMG amplitude measured during eccentric knee flexion MVC [as described previously (Trezise et al., 2016)]. For the eccentric MVCs, antagonist EMG was normalized to the concentric knee flexion MVC.

The interpolated twitch method was used to estimate percent voluntary activation during the isometric MVCs (Millet et al., 2003) using the equation: %VA = (1−superimposed twitch/potentiated twitch) × 100.

### Muscle Size and Architecture

**FIGURE 1:**
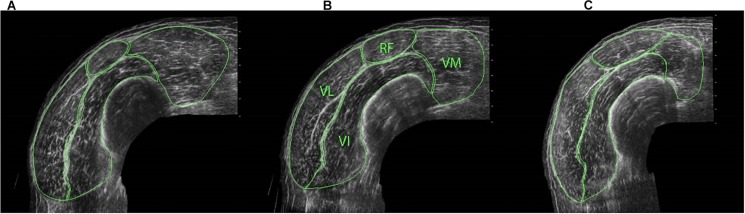
ACSA of individual quadriceps components at distal **(A)**, middle **(B)** and proximal **(C)** regions of the thigh, identifying rectus femoris (RF), vastus medialis (VM) vastus lateralis (VL) and vastus intermedius (VI).

Muscle anatomical cross-sectional area (CSA), muscle fascicle length (l_f_) and muscle fascicle angle (θ_f_) were obtained using two-dimensional B-mode ultrasonography (Aloka SSD-α10, software number 6.1.0, Aloka Co., Ltd., Tokyo, Japan) with a 10 MHz 60-mm linear-array transducer. Extended-field-of-view mode was used for all images (see Figure 1) (Noorkoiv et al., 2010a,b). Anatomical CSA was used to represent muscle size as it has both the benefit of enabling the analysis of region-specific differences in an individual, and being a good predictor of both isometric and isokinetic muscle force (Ahtiainen et al., 2003; Ikegawa et al., 2008; Blazevich et al., 2009). During measurements, participants lay relaxed with their legs fully extended in the supine position. To remove compression of the muscles a rolled towel was positioned behind the knee joint.

Cross-sectional area measurements were obtained at proximal (50%), middle (40%) and distal (30%) regions of the thigh [centre of the patella to the medial aspect of the anterior superior iliac spine (Noorkoiv et al., 2010a)] for the whole quadriceps and for each quadriceps component separately (i.e., RF, VL, VI, and VM) (Figure 1) to allow for between- and within-muscle variability in hypertrophy to be examined (Narici et al., 1996; Ahtiainen et al., 2003; Ema et al., 2013). When the separation of vastii muscles was not clear in the proximal images due to a lack of observable inter-muscular septum, a line was drawn from the end of the visible septum to a landmark on the muscle’s circumference that had been observable on the mid-muscle region images (Blazevich et al., 2007). For both CSA and fascicle measurements (see below), three scans were obtained at each location and the median value was used in the analysis. All ultrasound images were manually traced using ImageJ software (1.41o, National Institute of Health, United States). For these CSA measures, CVs ranged 2.9 ± 1.6 (distal) to 4.2 ± 3.0% (proximal) for RF, 2.1 ± 1.5 (mid) to 2.6 ± 1.4% (proximal) for VL, 1.3 ± 0.9 (mid) to 2.4 ± 1.4% (proximal) for VI, and 2.1 ± 1.5 (proximal) to 3.7 ± 2.8% (distal) for VM.

Fascicle length and angle measurements were also obtained from all four quadriceps components to account for the heterogeneity between and within the muscles (Blazevich et al., 2006). From the distance between the lateral border of the patella and greater trochanter, three sites were acquired along VL (33, 50 and 67% providing distal, middle and proximal regions), and one on VI (mid-region; lateral view). Images for VM were obtained from the 25% CSA site, and images for RF from the 50% CSA site mentioned above. Each measurement site was marked by 4-mm wide adhesive tape strip which provided a shadow in the ultrasound image. Fascicle length was defined as the distance between the superficial and deep aponeurosis of the fascicle that crossed the mid-point of the shadow and fascicle angle measurements were then obtained from the same fascicle. As the fascicles have slightly greater curvature at the deep aponeurosis insertion point, fascicle angle measurements were obtained from 3-mm above the deep aponeurosis to a line drawn 50% along the length of the fascicle (Blazevich et al., 2006). Due to the significant curvature of VM fascicles, VM fascicle length was measured from the fascicle that crossed 1/3 the distance between the superficial and deep aponeuroses, and VM fascicle angle measured from the deep aponeurosis for 2 cm along the length of the fascicle. CVs for FL ranged 1.7 ± 1.0 to 3.7 ± 2.1%, with the smallest obtained in VI and the largest in VL_DIST_, whilst CVs for FA ranged 1.7 ± 1.0 to 3.8 ± 2.5%, with the smallest obtained in VM and the largest in RF.

### Moment Arm Distance

The patellar tendon moment arm distance (MA) was obtained using seven sagittal-plane, low-radiation x-ray scans of the knee joint (Siemens Multi-MT 1384 model number 4803404). Participants lay supine with their knees flexed and their feet against a custom-built wooden frame. The seven knee joint angles (40, 50, 60, … 100°) were set using a hand-held goniometer. Due to a difference in moment arm measurements between relaxed and contracted states (Tsaopoulos et al., 2006), all scans were obtained with the participants performing isometric knee extension contractions against the foot plate at approximately 60% of MVC, which gave MA measurements similar to MVC (e.g., see Trezise et al., 2016).

The patellar tendon moment arm distance was measured as the perpendicular distance from the line of action of the tendon to the instantaneous centre of rotation (ICR) (Tsaopoulos et al., 2009). The ICR was located using the Reuleaux graphical analysis method (Maganaris et al., 1998; Tsaopoulos et al., 2009) with Photoshop software (Adobe Photoshop CS5, United States). To provide an estimate of the moment arm distance over the entire joint angle range, a third-order polynomial (41) (*R*^2^ > 0.90) was fitted to five measured moment arm distances from 50 to 90°. The inter-day reliability analysis (six participants over 3-sessions) across all five joint angles yielded a CV of 3.1 ± 2.0% (∼1.4°).

### Data Analysis

Five separate repeated measures multivariate analyses of variance (MANOVA), with time as the within-participant variable, were conducted to assess post-training changes in (1) isometric, concentric and eccentric peak knee extensor torque, and unpotentiated and potentiated twitch torques; (2) muscle activation (normalized EMG amplitudes); (3) M-wave amplitude measured during an isometric contraction; (4) quadriceps and individual muscle CSAs; and (5) fascicle angles and fascicle lengths in each muscle. When significant time effects were observed, additional ANOVAs or univariate analyses were performed as appropriate to determine the location of the change. Changes in percent voluntary activation and moment arm (moment arm at the angle of peak torque was considered changeable with training) were analyzed using paired *t*-tests. Normality of data distribution was confirmed using the Shapiro-Wilk test. Data that were not normally distributed (i.e., percent voluntary activation (%VA) and VM M-wave amplitude) were log transformed prior to statistical analysis. Multicollinearity was checked by computing correlations between input variables, with *r* < 0.8 being taken as a cut-off value. Analyses were performed using SPSS (version 20.0.0 IBM Corp., New York, NY, United States). Descriptive data are displayed as mean ± standard deviation in the text and tables, and as mean ± standard error of the mean (SE) in the figures. Significance was accepted at *p* ≤ 0.05. Although no overall correction was applied for the number of ANOVAs used, type I error rate inflation should be considered when interpreting data from the current analysis.

A set of multiple regression models were developed *a priori* to examine the relationships between the change in maximum torque (ΔT) and the changes in anatomical and neuromuscular variables (ΔVAR). The relative quality of the models was subsequently tested using Akaike’s Information Criterion (AIC), as described below. The predictor variables included in each model were considered to theoretically influence maximum torque production (Narici et al., 1996; Blazevich et al., 2009). Individual %VA data obtained during the isometric contractions were also included in the concentric and eccentric candidate model sets to allow an inference of maximal activation capacity. When assessing the change scores, an absolute change was considered a more important indicator of change than percentage change, as a similar relative change would require large improvements by stronger, and only small improvements by weaker, individuals. Muscle activation, however, was quantified as the percent change in order to minimize the influence of individual variability in EMG resulting from anatomical differences (e.g., adipose tissue thickness). Additionally, as a significant change in moment arm following training would only result from a change in the knee joint angle at which maximum torque is produced, and total moment arm distance is important for the amplification of muscle force production, moment arm distance measured before training was included in the models.

To examine the effect of specific clusters of variables, a multi-step approach was taken. First, scatter plots were constructed to identify the relationships between ΔVAR and ΔT for each contraction mode. When the relationship between ΔVAR (or pre-training moment arm) and ΔT appeared to be non-linear the nature of the relationship was identified using polynomial curve fitting, with curve order being increased until the change in R^2^ was less than 2% (Waugh et al., 2012). These variables were added as non-linear data in the models (combined with the linear variables). The distributions of the dependent variables were checked for normality and both the changes in isometric (ΔT_ISO_) and eccentric (ΔT_ECC_) torque were transformed using the natural log due to non-normal distributions. Correlations were then computed to assess the isolated relationships between the changes in the anatomical and neuromuscular variables and the change in maximal torque for each contraction mode. Where the strength of the correlation has been interpreted, Cohen’s standards have been used.

The best model for each contraction mode was selected based on Akaike’s Information Criterion (AIC) (Burnham and Anderson, 2002; Arnold, 2010), as noted previously (Trezise et al., 2016). The models contained within the candidate model set for each contraction mode were all considered *a priori* to be theoretically influential to maximal torque production. To rank the models, the AIC adjusted for small sample size (AIC_C_) was used (Burnham and Anderson, 2002). The model with the lowest AIC_C_ value was considered the best fit for that strength measure, and all models with ΔAIC_C_ ≤ 2 were considered to have substantial support (Burnham and Anderson, 2002). The Akaike Weights (*w*_i_) sum to one, and classify the probability of each model being the best-ft model within that candidate model set, with those with a higher likelihood having a greater weight (Wagenmakers and Farrell, 2004). Between 22 and 25 models were developed for each contraction mode, with combinations of variables determined by both their theoretical likelihood of influencing the change in torque and on the strength of their individual correlations with the changes in torque. Adjusted *R*^2^ values were used in combination with the AIC_C_ rankings to identify the percentage of torque that could be explained by the models.

To determine whether the neuromuscular variables previously identified as the best predictors from cross-sectional analysis were the same variables deemed to influence the change in torque following training, the predictors from the ‘best-fit’ model for each contraction mode from our previous cross-sectional study (Trezise et al., 2016) were also correlated with ΔT. All regression models were analyzed using R version 3.0.0 (R Core Team, 2013).

## Results

All participants increased in strength following the 10-week training period (see Table 1). Maximal isometric, concentric, eccentric torques increased by 17.2 ± 12.6%, 12.5 ± 8.0% and 16.2 ± 14.4%, respectively (*p* < 0.01 for all; Table 1). These changes were less than the 46.6 ± 21.0% increase in 6-RM knee extension strength across the training period (data not shown). Strong correlations (*r* = 0.73–0.78) were observed between their final maximum leg extension load and maximum torque for each contraction mode following training, however, weak or no correlations (*r* = 0.07–0.25) were observed between the change in maximum leg extension load and the change in maximum torque for each contraction mode. Changes were observed in a number of variables relating to muscle activation, but not co-activation or moment arm at the angle of peak torque after training (Table 1). Statistically significant increases were also observed for all CSA, fascicle angle and fascicle length measurements, as shown in Table 2. Participants who produced greater torque at pre-training were equally likely to increase absolute strength as the weaker participants, as demonstrated by strong correlations between pre- and post-training torque values (*r* = 0.93, 0.97 and 0.90, all *p* ≤ 0.001, for isometric, concentric and eccentric torque, respectively).

**TABLE 1:** Training loads, and torque, moment arm, and muscle activity and activation variables obtained before and after training during maximal isometric, and isokinetic concentric and eccentric contractions.

**Variable**	**Pre-training (Mean ± SD)**	**Post-training (Mean ± SD)**	**Absolute change (Mean ± SD)**	**Percent change (Mean ± SD)**
**Training Load: 6-RM**				
Knee extension (kg)	76.2 ± 18.5	109.6 ± 25.8	33.4 ± 15.2	45.8 ± 19.7
Leg press (kg)	134.7 ± 48.1	275.0 ± 91.3	140.2 ± 62.1	114.2 ± 58.6
Leg curl (kg)	42.9 ± 7.8	62.6 ± 10.6	19.7 ± 7.4	47.6 ± 19.7
**Isometric**				
Torque_ISO_ (N⋅m)	256.4 ± 69.1	296.8 ± 74.3	39.7 ± 25.6^**^	17.2 ± 12.6
T_Un–Tw_ (N⋅m)	48.0 ± 13.6	48.6 ± 12.9	0.5 ± 11.4	6.9 ± 42.2
T_Pot–Tw_ (N⋅m)	65.8 ± 19.42	70.29 ± 16.8	4.5 ± 15.0	14.4 ± 48.4
MA (mm)	50 ± 5	50 ± 4	−0.3 ± 1.9	−0.6 ± 3.8
M-Wave_RF_ (mV)	4.31 ± 1.31	4.05 ± 1.33	−0.3 ± 1.0	−4.5 ± 22.9
M-Wave_VL_ (mV)	5.09 ± 1.58	4.91 ± 1.60	−0.2 ± 1.6	2.0 ± 42.0
M-Wave_VM_ (mV)	3.06 ± 2.13	2.54 ± 1.32	−0.5 ± 2.6	0.8 ± 51.4
EMG:M_AVEQ_	0.075 ± 0.023	0.089 ± 0.022	−	23.2 ± 25.5^**^
EMG:M_RF_	0.066 ± 0.020	0.083 ± 0.025	−	33.8 ± 42.9^**^
EMG:M_VL_	0.072 ± 0.028	0.084 ± 0.027	−	24.4 ± 35.0^**^
EMG:M_VM_	0.087 ± 0.038	0.099 ± 0.040	−	23.7 ± 45.3^*^
EMG_BF_	0.244 ± 0.09	0.226 ± 0.08	−	−5.1 ± 26.7
%VA (%)	88.51 ± 6.71	92.00 ± 4.99	−	4.1 ± 3.6^**^
**Concentric**				
Torque_CON_ (N⋅m)	223.5 ± 61.5	248.2 ± 60.9	24.7 ± 13.9^**^	12.5 ± 8.0
MA (mm)	50 ± 5	49 ± 5	0.17 ± 1.8	−0.4 ± 3.7
EMG:M_AVEQ_	0.081 ± 0.020	0.094 ± 0.025	−	19.0 ± 26.4^**^
EMG:M_RF_	0.083 ± 0.031	0.092 ± 0.035	−	14.8 ± 34.9
EMG:M_VL_	0.071 ± 0.022	0.086 ± 0.033	−	21.9 ± 38.4^**^
EMG:M_VM_	0.089 ± 0.037	0.111 ± 0.050	−	23.4 ± 36.8^**^
EMG_BF_	0.257 ± 0.10	0.272 ± 0.12	−	10.9 ± 38.5
**Eccentric**				
Torque_ECC_ (N⋅m)	274.5 ± 73.6	315.8 ± 73.8	40.4 ± 32.0^**^	16.2 ± 14.4
MA (mm)	48 ± 5	48 ± 5	−0.2 ± 1.8	−0.4 ± 3.7
EMG:M_AVEQ_	0.066 ± 0.019	0.080 ± 0.024	−	22.7 ± 28.4^**^
EMG:M_RF_	0.067 ± 0.026	0.078 ± 0.030	−	21.6 ± 39.2^*^
EMG:M_VL_	0.062 ± 0.023	0.077 ± 0.030	−	27.0 ± 36.5^**^
EMG:M_VM_	0.068 ± 0.031	0.090 ± 0.046	−	23.3 ± 37.4^*^
EMG_BF_	0.226 ± 0.10	0.227 ± 0.10	−	5.7 ± 33.8

Although data were entered into ANOVAs as absolute data (excluding muscle activation variables), percent changes (±SD) are also presented for comparison. Torque variables include maximum voluntary torque (Torque_*ISO/CON/ECC*_) as well as unpotentiated (T_*Un*–*Tw*_) and potentiated (T_*Pot*–*Tw*_) twitch torques. Muscle activation variables include peak-to-peak M-Wave amplitude of rectus femoris (RF), vastus lateralis (VL) and vastus medialis (VM); EMG:M, normalized average quadriceps (EMG:M_*AVEQ*_), RF (EMG:M_*RF*_), VL (EMG:M_*VL*_) and VM (EMG:M_*VM*_) EMG amplitudes; EMG_*BF*_ = biceps femoris EMG amplitude normalized to EMG during MVC; MA, patella tendon moment arm measured at the angle of peak isometric, concentric or eccentric torque; %VA, percent voluntary activation. ^*^p ≤ 0.05; ^∗∗^*p* ≤ 0.01.

**TABLE 2:** Muscle size, fascicle angle, and fascicle length obtained before and after training.

**Variable**	**Pre-training (Mean ± SD)**	**Post-training (Mean ± SD)**	**Absolute Change (Mean ± SD)**	**Percentage Change (Mean ± SD)**
CSA,Q_PROX_ (cm^2^)	76.3 ± 14.1	80.0 ± 14.3	3.7 ± 2.6^**^	4.9 ± 3.4
CSA,Q_MID_ (cm^2^)	72.6 ± 15.3	77.9 ± 15.3	5.2 ± 2.9^**^	7.3 ± 4.0
CSA,Q_DIST_ (cm^2^)	60.6 ± 11.9	66.0 ± 13.7	5.3 ± 3.8^**^	9.0 ± 6.3
CSA,Q_SUM_ (cm^2^)	209.5 ± 40.9	223.7 ± 42.5	14.2 ± 8.4^**^	7.0 ± 4.3
CSA,RF_PROX_ (cm^2^)	9.6 ± 2.4	10.6 ± 2.6	0.9 ± 0.8^**^	9.7 ± 8.2
CSA,RF_MID_ (cm^2^)	5.9 ± 1.8	6.7 ± 2.1	0.7 ± 0.5^**^	12.1 ± 8.8
CSA,RF_DIST_ (cm^2^)	3.0 ± 1.1	3.5 ± 1.3	0.5 ± 0.4^**^	16.7 ± 14.7
CSA,RF_SUM_ (cm^2^)	18.6 ± 4.9	20.8 ± 5.6	2.2 ± 1.5^**^	11.7 ± 7.1
CSA,VL_PROX_ (cm^2^)	24.7 ± 5.6	26.3 ± 5.6	1.6 ± 1.5^**^	6.6 ± 5.9
CSA,VL_MID_ (cm^2^)	21.8 ± 5.7	24.2 ± 5.9	2.4 ± 1.8^**^	11.2 ± 8.4
CSA,VL_DIST_ (cm^2^)	15.2 ± 3.8	17.3 ± 4.2	2.1 ± 1.5^**^	13.6 ± 9.7
CSA,VL_SUM_ (cm^2^)	61.6 ± 14.5	67.6 ± 15.2	6.0 ± 3.7^**^	10.2 ± 6.8
CSA,VI_PROX_ (cm^2^)	30.4 ± 5.9	31.9 ± 6.3	1.5 ± 1.6^**^	5.1 ± 5.2
CSA,VI_MID_ (cm^2^)	26.4 ± 6.0	28.6 ± 6.2	2.2 ± 1.6^**^	8.3 ± 5.5
CSA,VI_DIST_ (cm^2^)	19.8 ± 4.3	21.7 ± 5.0	1.5 ± 1.5^**^	7.4 ± 7.5
CSA,VI_SUM_ (cm^2^)	76.7 ± 15.7	82.3 ± 16.9	5.6 ± 3.7^**^	7.4 ± 4.4
CSA,VM_PROX_ (cm^2^)	8.7 ± 2.2	9.5 ± 2.4	0.7 ± 0.8^**^	8.4 ± 8.8
CSA,VM_MID_ (cm^2^)	15.4 ± 3.3	16.6 ± 3.3	1.3 ± 1.0^**^	8.4 ± 6.5
CSA,VM_DIST_ (cm^2^)	20.1 ± 3.8	21.4 ± 3.6	1.4 ± 1.2^**^	6.9 ± 5.9
CSA,VM_SUM_ (cm^2^)	44.1 ± 8.4	47.5 ± 8.5	3.4 ± 2.3^**^	8.2 ± 6.3
θ_f_VL_PROX_ (°)	19.6 ± 4.1	20.4 ± 4.0	0.9 ± 1.4^**^	4.6 ± 7.1
θ_f_VL_MID_ (°)	17.6 ± 4.0	18.4 ± 3.3	0.9 ± 2.1^*^	4.9 ± 12.1
θ_f_VL_DIST_ (°)	17.8 ± 3.5	18.6 ± 3.3	0.8 ± 2.2^*^	4.5 ± 12.5
θ_f_RF (°)	14.1 ± 3.7	15.1 ± 3.0	1.0 ± 1.7^**^	6.7 ± 12.3
θ_f_VI (°)	14.1 ± 3.6	14.7 ± 3.5	0.7 ± 1.8^*^	4.6 ± 12.7
θ_f_VM (°)	36.8 ± 3.7	38.6 ± 4.2	1.8 ± 2.5^**^	5.0 ± 6.8
ℓ_f_VL_PROX_ (cm)	7.7 ± 1.1	8.0 ± 1.0	0.3 ± 0.6^**^	3.9 ± 7.4
ℓ_f_VL_MID_ (cm)	7.8 ± 137	8.2 ± 1.4	0.4 ± 0.7^**^	4.5 ± 9.2
ℓ_f_VL_DIST_ (cm)	7.6 ± 1.3	8.2 ± 1.5	0.6 ± 0.7^**^	7.4 ± 8.5
ℓ_f_RF (cm)	9.0 ± 1.9	9.3 ± 1.8	0.3 ± 0.7^**^	3.6 ± 7.4
ℓ_f_VI (cm)	7.4 ± 1.4	7.8 ± 1.4	0.5 ± 0.7^**^	6.1 ± 9.0
ℓ_f_VM (cm)	9.0 ± 1.0	8.6 ± 1.0	0.3 ± 0.5^**^	2.8 ± 5.9

Although muscle size and architecture data were entered into ANOVAs as absolute data, percent changes (± SD) are also presented for comparison. CSA, cross sectional area of the quadriceps (Q), rectus femoris (RF), vastus lateralis (VL), vastus intermedius (VI), and vastus medialis (VM). θ_*f*_, fascicle angle, ℓ_*f*_, fascicle length. PROX, MID and DIST refer the proximal, mid-muscle and distal regions of the thigh; SUM, total of all CSA regions for that quadriceps measure. ^*^p ≤ 0.05, ^∗∗^*p* ≤ 0.01.

### Regression Models

#### Change in Torque Versus the Change in Anatomical and Neuromuscular Variables

**FIGURE 2:**
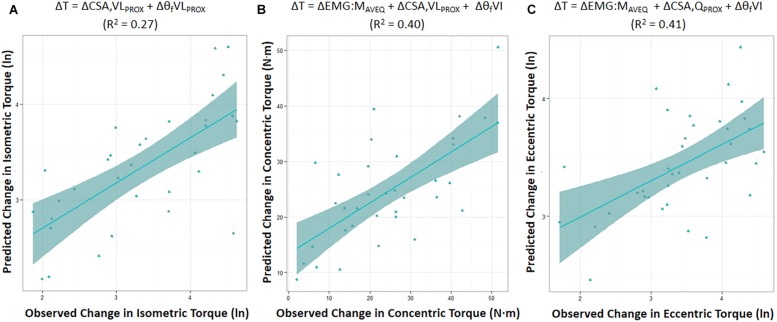
Predicted change in torque (ΔT) was modeled based on the AIC_C_ rankings using the best-fit model for the change in maximal isometric **(A)**, and isokinetic concentric **(B)** and eccentric **(C)** torque prediction. Figures show the mean (± SE) for each model. (ln) = the natural log of the change in torque. CSA,Q_PROX_ and CSA,VL_PROX_ = proximal cross-sectional area of whole quadriceps, or of vastus lateralis (VL) in isolation, respectively; EMG:M_AVEQ_ = normalized average quadriceps (AVEQ) amplitude; θ_f_VL_PROX_ and θ_f_VI = fascicle angle of VL obtained at proximal region, and vastus intermedius (VI), respectively; *R*^2^ = adjusted *R*^2^.

Moderate relationships were observed between the best-fit models and the changes in torque for all contraction modes (Table 3). The best-fit model for the change in isometric torque was ‘ΔCSA,VL_PROX_ + Δθ_f_VL_PROX_’ (*R*^2^ = 0.27, AIC_c_*w*_i_ = 0.52) while the best-fit models for the change in concentric and eccentric torques were ‘ΔEMG:M_AVEQ_ + ΔCSA,VL_PROX_ + Δθ_f_VI’ (*R*^2^ = 0.40, AIC_c_*w*_i_ = 0.15) and ‘ΔEMG:M_RF_ + ΔCSA,Q_PROX_ + Δθ_f_VI’ (*R*^2^ = 0.41, AIC_c_*w*_i_ = 0.31), respectively (Table 3). Models incorporating the change in mid-region vastus lateralis fascicle length (l_f_VL_MID_) or angle (θ_f_VL_MID_) also had substantial support for inclusion in the concentric torque prediction models, and the change in percent voluntary activation (%VA) and pre-training moment arm distance had substantial support (AIC_C_ < 2) for use in the eccentric models (Table 4). Based on the best-fit models for each contraction mode, the mean (± SE) absolute errors in the prediction of the change in torque were 16.1 ± 3.1% (isometric), 59.8 ± 12.9% (concentric) and 17.6 ± 2.3% (eccentric) (see Figure 2).

**TABLE 3:** The best-fit model for predicting changes in maximum isometric and isokinetic concentric and eccentric torque (ΔT) from the changes in predictor variables (ΔVAR).

**Contraction**	**Best-fit Model**	**Equation**	***R*^2^**
Δ*T*_ISO_	**ΔCSA,VL_PROX_ + Δθ_f_VL_PROX_**	Y = 0.210(ΔCSA,VL_PROX_) + 0.199(Δθ_f_VL_PROX_) + 2.924	0.27
Δ*T*_CON_	**ΔEMG:M_AVEQ_ + ΔCSA,VL_PROX_ + Δθ_f_VI**	Y = 0.251(ΔEMG:M_AVEQ_) + 2.453 (ΔCSA,VL_PROX_) + 2.537(Δθ_f_VI) + 14.633	0.40
Δ*T*_ECC_	**ΔEMG:M_AVEQ_ + ΔCSA,Q_PROX_ + Δθ_f_VI**	Y = −0.124(ΔCSA,Q_PROX_) + 0.016(ΔEMG:M_AVEQ_) + 0.170(Δθ_f_VI) + 3.334	0.41

*For each candidate model: θ_*f*_, fascicle angle of proximal vastus lateralis (VL_*PROX*_) or vastus intermedius (VI); CSA, proximal-region vastus lateralis (CSA,VL_*PROX*_) or whole quadriceps (CSA,Q_*PROX*_) cross-sectional area of whole; ΔEMG:M_*AVEQ*_, amplitude of average quadriceps normalized to the M-wave; EMG:M is represented as the percentage change while the other predictor variables are represented as an absolute change. *R*^2^, adjusted *R*^2^.*

**TABLE 4:** Akaikes’ Information Criterion (AIC) of model parameters for predicting the change in isometric, concentric and eccentric torque (ΔT) based on changes in the predictor variables (Δ).

***Contraction***	**Model**	**K**	**AIC_C_**	**ΔAIC_C_**	**AIC_C_*w*_i_**	**LL**	**R^2^**
*ln (*Δ*T*_ISO)_							
	ΔCSA,VL_PROX_ + Δθ_f_VL_PROX_	4	77.48	0.00	0.52	–34.10	0.27
	ΔCSA,VL_PROX_ + Δθ_f_VL_PROX_ + Δ%VA	5	79.80	2.32	0.16	–33.90	0.25
	ΔCSA,VL_PROX_ + Δθ_f_VL_PROX_ + MA	5	80.08	2.60	0.14	–34.04	0.25
	ΔCSA,VL_PROX_	3	81.20	3.72	0.08	–37.22	0.15
	ΔCSA,Q_PROX_ + Δθ_f_VL_PROX_	4	81.29	3.81	0.08	–36.00	0.18
	Intercept Only	2	85.82	8.34	0.01	–40.73	−
Δ*T*_CON_							
	ΔEMG:M_AVEQ_ + ΔCSA,VL_PROX_ + Δθ_f_VI	5	273.35	0.00	0.15	–130.64	0.40
	ΔEMG:M_AVEQ_ + ΔCSA,VL_PROX_ + Δl_f_VL_MID_	5	273.91	0.56	0.11	–130.92	0.40
	ΔEMG:M_AVEQ_ + Δθ_f_VI	4	274.01	0.65	0.11	–132.34	0.36
	ΔEMG:M_AVEQ_ + ΔCSA,Q_PROX_ + Δθ_f_VI	5	274.55	1.20	0.08	–131.24	0.38
	ΔEMG:M_RF_ + ΔCSA,Q_PROX_ + Δθ_f_VI	5	274.55	1.20	0.08	–131.24	0.38
	ΔEMG:M_AVEQ_ + ΔCSA,Q_PROX_ + Δl_f_VL_MID_	5	274.55	1.20	0.08	–131.24	0.38
	ΔEMG:M_AVEQ_ + ΔCSA,VL_PROX_ + Δθ_f_VL_MID_	5	274.68	1.33	0.08	–131.31	0.37
	ΔEMG:M_RF_ + Δθ_f_VI	4	275.01	1.66	0.07	–132.84	0.36
	Intercept Only	2	287.05	13.70	0.00	–141.34	−
*ln (*Δ*T_ECC_)*							
	ΔEMG:M_AVEQ_ + ΔCSA,Q_PROX_ + Δθ_f_VI	5	75.84	0.00	0.30	–31.77	0.41
	ΔEMG:M_RF_ + ΔCSA,Q_PROX_ + Δθ_f_VI + Δ%VA	6	77.06	1.22	0.16	–30.85	0.43
	ΔEMG:M_AVEQ_ + ΔCSA,Q_PROX_ + Δθ_f_VI + MA	6	77.55	1.71	0.13	–31.09	0.42
	ΔEMG:M_AVEQ_ + ΔCSA,Q_PROX_ + Δθ_f_VI + Δ%VA	6	77.63	1.79	0.12	–31.14	0.42
	ΔEMG:M_RF_ + ΔCSA,Q_PROX_ + Δθ_f_VI	5	78.23	2.39	0.09	–32.96	0.37
	Intercept Only	2	88.35	12.51	0.00	–41.97	−

*Models with both substantial support (ΔAIC_*c*_ ≤ 2) for predicting the change in torque, and an AIC_*C*_*w*_*i*_ ≥ 0.10 (i.e., greater than a 10% chance that they will be the best-fit model), are identified by shading. ln refers to the natural log of the ΔT. For each candidate model: CSA,Q_*PROX*_ and CSA,VL_*PROX*_, proximal cross-sectional area of whole quadriceps, or of vastus lateralis (VL) in isolation, respectively; θ_*f*_VL_*PROX*_ and θ_*f*_VI, fascicle angle of VL obtained at the proximal region, and vastus intermedius (VI), respectively; Δl_*f*_VL_*MID*_, fascicle length of VL obtained at the middle region; ΔEMG:M_*AVEQ*_ and ΔEMG:M_*RF*_, amplitude of normalized average quadriceps (AVEQ) or rectus femoris (RF) EMG:M amplitude, respectively; %VA, percent voluntary activation (obtained during isometric contractions); MA, patella tendon moment arm distance. Intercept, basic control model with no predictors, and includes only the constant and residual variance (σ^2^). EMG is represented as a percent change; the other predictor variables are represented as an absolute change aside from MA, which is included as the pre-training moment arm distance. K, number of parameters tested in each model; AIC_C_, Akaike’s information criterion for a small data set; ΔAIC_*C*_, the models AIC_*C*_ minus the minimum AIC_*C*_ among candidate models. AIC_*C*_*w*_*i*_, the percentage of times that a given model would be selected as the “best model” by AIC_*C*_, and serves as the weight of evidence for a given model being the best model from that set of candidate models (Burnham and Anderson, 2002); *R*^2^, adjusted *R*^2^.*

While fascicle angle was present in all best-fit models, Δθ_f_VL_PROX_ appeared in the isometric torque prediction models whereas Δθ_f_VI appeared in the concentric and eccentric torque models. Similarly, the change in VL CSA was included in the best-fit isometric and concentric torque models (ΔCSA,VL_PROX_), while whole quadriceps CSA measured proximally (ΔCSA,Q_PROX_) was included in the eccentric torque prediction models. There was also substantial support for models incorporating the percent changes for both the average quadriceps (ΔEMG:M_AVEQ__)_ and rectus femoris (ΔEMG:M_RF_) muscle activation variables for both concentric and eccentric torque production. Models incorporating the change in antagonist EMG (EMG_BF_) and unpotentiated twitch torque did not have enough support to be included in the final candidate model set for any contraction mode.

#### Change in Torque Verses the Change in the “Best-Fit” Parameters Identified From Cross-Sectional Analysis

In a previous study (Trezise et al., 2016) we determined the combination of neuromuscular variables that best explained an individual’s maximum strength using a cross-sectional study design. The specific variables in those models were also tested in the present study to examine whether the models that could explain the greatest variance in maximum torque production on a cross-sectional basis could also explain a significant proportion of the variance in the change in torque over a period of training, however, no relationship was observed for any contraction mode (*R*^2^ = 0.00 to 0.07; Table 5).

**TABLE 5:** Regression models using the previously identified “best-fit” model parameters for predicting maximal torque in a cross-sectional analysis (Trezise et al., 2016) to determine whether adaptations in these variables were associated with changes in strength following training.

**Contraction**	**Model**	**R^2^**
		
Δ*T*_ISO_	ΔCSA,Q_PROX_ + ΔEMG:M_AVEQ_ + Δθ_f_VL_MID_ + Δ%VA	0.07
Δ*T*_CON_	ΔCSA,Q_PROX_ + Δθ_f_VL_PROX_ + MA	0.00

*CSA_*PROX*_, proximal cross-sectional area; ΔEMG: M, amplitude of average quadriceps (AVEQ) normalized to the M-wave; %VA, percent voluntary activation; MA, patella tendon moment arm obtained pre-training. EMG: M is represented as the percentage change while the other predictor variables are represented as an absolute change. *R*^2^, adjusted *R*^2^.*

### Correlations

#### Correlations Between Changes in Torque and Changes in Muscle Activation Variables

While the percent changes in agonist muscle activation variables were not correlated with the change in isometric torque, ΔEMG:M_AVEQ_ and ΔEMG:M_RF_ were moderately correlated with the change in both concentric (*r* = 0.52, *p* < 0.01; *r* = 0.56, *p* < 0.001) and eccentric (*r* = 0.56, *p* < 0.001; *r* = 0.51, *p* < 0.01) torques, as shown in Table 6. ΔEMG:M_VM_ was also weakly correlated with the change in both concentric (*r* = 0.35, *p* < 0.05) and eccentric (*r* = 0.48, *p* < 0.01) torque.

**TABLE 6:** Correlations (r) between the change in isometric (ΔT_ISO_), and isokinetic concentric (ΔT_CON_) and eccentric (ΔT_ECC_) torque and changes in neuromuscular variables (ΔVAR).

**ΔVAR**	**ΔT_ISO_**	**ΔT_CON_**	**ΔT_ECC_**
%Δ EMG:M_AVEQ_	0.17	0.52^∗∗^	0.56^∗∗∗^
%Δ EMG:M_RF_	0.10	0.56^∗∗∗^	0.51^∗∗^
%Δ EMG:M_VL_	0.00	0.28	0.31
%Δ EMG:M_VM_	0.11	0.35^*^	0.48^∗∗^
%Δ EMG_BF_	0.00	0.10	0.20
Δ%VA	0.23	0.08	0.17
MA	0.13	0.11	–0.10
ΔCSA,Q_PROX_	0.36^*^	0.23	–0.26
ΔCSA,Q_MID_	0.30	0.07	–0.26
ΔCSA,Q_DIST_	0.29	0.20	0.17
ΔCSA,RF_PROX_	0.32	0.00	0.04
ΔCSA,RF_MID_	0.20	0.04	0.06
ΔCSA,RF_DIST_	0.26	0.23	0.15
ΔCSA,VL_PROX_	0.42^*^	0.35^*^	0.09
ΔCSA,VL_MID_	0.45^∗∗^	0.03	0.03
ΔCSA,VL_DIST_	0.26	0.16	0.12
ΔCSA,VI_PROX_	0.30	0.36	0.04
ΔCSA,VI_MID_	0.29	0.17	0.00
ΔCSA,VI_DIST_	0.36^*^	0.08	0.12
ΔCSA,VM_PROX_	–0.18	0.12	–0.05
ΔCSA,VM_MID_	0.12	0.06	–0.26
ΔCSA,VM_DIST_	0.11	0.07	–0.29
Δθ_f_VL_PROX_	0.41^*^	0.00	0.16
Δθ_f_VL_MID_	0.16	0.26	0.06
Δθ_f_VL_DIST_	0.13	0.15	0.10
Δθ_f_RF	0.19	–0.06	0.23
Δθ_f_VI	0.05	0.39^*^	0.26
Δθ_f_VM	0.27	0.28	–0.10
Δl_f_VL_PROX_	0.13	–0.20	–0.18
Δl_f_VL_MID_	–0.06	−0.34^*^	–0.15
Δl_f_VL_DIST_	–0.12	0.17	0.00
Δl_f_RF	–0.27	–0.22	−0.43^*^
Δl_f_VI	0.00	0.13	–0.03
Δl_f_VM	0.23	–0.05	–0.09

Be mindful that to assess the relationship between these neuromuscular variables involved and all three maximal contractions modes, a large number of correlations are calculated. At the significance level of *p* = 0.05, 1 in 20 of these correlations can be expected by chance alone. EMG: M, normalized average quadriceps (AVEQ), rectus femoris (RF), vastus lateralis (VL), vastus medialis (VM) amplitude; EMG_*BF*_, amplitude MA, patella tendon moment arm; %VA, percent voluntary activation. CSA, cross sectional area of the whole quadriceps (Q), rectus femoris (RF) vastus lateralis (VL), vastus intermedius (VI), and vastus medialis (VM). θ_*f*_, fascicle angle, l_*f*_, fascicle length. PROX, MID and DIST refer the proximal mid-muscle and distal regions of the thigh. Δ, absolute change;%Δ, percent change. ^*^p ≤ 0.05; ^∗∗^*p* ≤ 0.01; ^∗∗∗^*p* ≤ 0.001.

#### Correlations Between Changes in Torque and Changes in Muscle Size and Architecture

Changes in muscle size were more strongly correlated with the change in isometric torque than either concentric or eccentric torque. The change in proximal whole quadriceps CSA (ΔCSA,Q_PROX_) and the change in proximal (ΔCSA,VL_PROX_) and mid-region (ΔCSA,VL_MID_) VL CSA were weakly correlated with the change in isometric torque (*r* = 0.36 and 0.42, *p* < 0.05) and (*r* = 0.45, *p* < 0.01), respectively; see Table 6. ΔCSA,VL_PROX_ was also weakly correlated with the change in concentric torque (*r* = 0.35, *p* < 0.05). The change in eccentric torque was not correlated with changes in any muscle size variable.

The change in proximal region VL FA (Δθ_f_VL_PROX_) was moderately correlated with the change in isometric torque (*r* = 0.41, *p* < 0.05) and the change in VI FA (Δθ_f_VI) was moderately correlated with the change in concentric torque (*r* = 0.41, *p* < 0.05); Table 6. The change in mid-region VL fascicle length was moderately correlated with the change in concentric (*r* = 0.35, *p* < 0.05), and the change in RF fascicle length was moderately correlated with the change in eccentric (*r* = 0.43, *p* < 0.05) torque. This change in fascicle length was the only muscle-based variable found to correlate with the change in eccentric torque following training.

## Discussion

Whilst muscle size, activation and architecture are considered to be important variables influencing maximum muscular force production (Narici et al., 1996) there is surprisingly little information regarding the relationship between changes in these variables and changes in strength following training. The present study examines the relationship between changes in isometric, concentric and eccentric knee extension strength and changes in specific anatomical and neuromuscular variables (i.e., muscle size, activation and architecture) following chronic (10 weeks) heavy (6-RM) strength training. The main conclusions are that (1) the change in isometric strength was moderately associated with changes in muscle size and fascicle angle; (2) the change in agonist muscle activation was the strongest predictor of the changes in maximum concentric and eccentric torque production (*r* = 0.51–0.56), and this relationship was strengthened when muscle size and fascicle angle were added to the predictive models; (3) pre-training moment arm distance and the change in percent voluntary activation (%VA) also appeared to influence the change in eccentric torque as they were included in models that received substantial support; (4) the best models previously identified for predicting maximum torque within a population (i.e., ‘CSA,Q_PROX_ + EMG:M_AVEQ_ + θ_f_VL_MID_ + %VA’ and ‘CSA,Q_PROX_ + θ_f_VL_PROX_ + MA’ for maximum isometric and concentric strength, respectively; (Trezise et al., 2016) were unable to predict the changes in torque with chronic training (*r* = 0.00–0.07); and (5) overall, 27–41% of the variance in the change in isometric, concentric and eccentric torques could be predicted by the changes in the anatomical and neuromuscular variables measured in the present study.

The four best candidate models for each contraction mode were ranked using Akaike’s information criterion (AIC_C_), an information-theoretic approach for model selection that determines the best-fit model by accounting for the goodness-of-fit of a model (i.e., the difference between the expected and the observed data) in conjunction with its simplicity (i.e., the number of variables included) (Burnham and Anderson, 2002; Arnold, 2010). The models were designed to predict the absolute changes in strength rather than the relative change to ensure that the influence of stronger participants was not reduced if their *relative* changes were modest compared to the weaker participants. *Post hoc*, the data analysis was repeated using relative change scores and, interestingly, little difference in outcome was observed (data not shown) so the information presented herein appears equally applicable to relative changes in strength. The training elicited strength increases (12.5–17.2%, see Table 1) that were similar in magnitude to those reported previously following similar-duration heavy strength training interventions (Braith et al., 1989; Rutherford and Jones, 1986; Ahtiainen et al., 2003; Balshaw et al., 2017). Nonetheless, although strength changes in all contraction modes were substantial and statistically significant, they were also highly variable between individuals (see large SD in Table 1). The changes in strength were slightly less than the 33.9 ± 15.7 kg (46.6 ± 21.0%; *p* < 0.001) increase in 6-RM knee extension strength following training. This discrepancy may have resulted from the different contraction modes performed in the training (isoinertial) and testing (isometric and isokinetic). The neuromuscular adaptations were measured during isometric and isokinetic contractions, as isoinertial training exercises also require greater activation of the stabilizing and synergist muscles and thus maximum quadriceps force production may be limited by strength and activation of the stabilizer muscles (Rutherford and Jones, 1986).

The best-fit models were found to explain 27, 40, and 41% of the inter-individual variation in the change in maximal isometric, concentric, and eccentric torque, respectively (Table 3). Model use led to mean (± SE) absolute errors in the prediction of the change in torque of 16.1 ± 3.1% (isometric), 59.8 ± 12.9% (concentric) and 17.6 ± 2.3% (eccentric) (Figure 2). Therefore, while changes in the anatomical and neuromuscular variables assessed in the present study appeared to be moderately associated with the change in maximum knee extension torque production (i.e., strength) following the 10-wk strength training period, the change in concentric torque in particular was poorly predicted. Thus, whilst the isometric and eccentric models can provide a reasonable estimate of maximal joint torque, care should be taken when using the concentric torque prediction model. Also, whilst measurement errors will reduce the explained variance in such models, ensuring that the values (27, 40, and 41%) somewhat under-represent the true capacity to predict strength change, the results also indicate that (i) the mechanisms influencing strength change must differ between individuals and (ii) mechanisms additional to those measured in the present study must have influenced the strength changes (see discussion below).

### Factors Associated With the Change in Isometric Torque

The best-fit model for the change in isometric torque included both the change in proximal VL CSA (vastus lateralis CSA) and the change in proximal VL fascicle angle. While the model explained only 27% of the change in isometric knee extension torque, it was the strongest model in the candidate set with an AIC_c_ weight (AIC_c_*w*_i_) of 0.57, indicating that 57% of the time the candidate model would be the best-fit model amongst that set of candidate models. The inclusion of CSA in the models is not surprising given that muscle size is considered to be a significant variable influencing joint torque production (Maughan et al., 1983; Schantz et al., 1983; Castro et al., 1995; Moss et al., 1997; Blazevich et al., 2009), and cross-sectional analyses show moderate-to-strong correlations between maximal voluntary strength and measures of muscle size (Maughan et al., 1983; Fukunaga et al., 2001; Blazevich et al., 2009; Erskine et al., 2010; Trezise et al., 2016). However, the relationship between the change in joint torque and the change in muscle size is not as clear. Of the few studies to assess this relationship following dynamic training in the quadriceps, weak-moderate correlations (*r* = 0.46 and 0.52) have been observed between the change in muscle size and the changes in isometric (Erskine et al., 2010, 2014; Balshaw et al., 2017) and eccentric (Higbie et al., 1996) strength, and a moderate correlation (*r* = 0.70) was found with the change in concentric (Higbie et al., 1996) strength. Higbie et al. (Higbie et al., 1996) speculated that the weaker relationship between the changes in muscle size and strength is unsurprising given that whole muscle CSA does not reflect the activation of muscle fibers, or the velocity-dependent nature of this activation. Interestingly, when accounting for region-specific hypertrophy, strong relationships have been observed between the change in proximal VL and isometric force developed at short muscle lengths (*r* = 0.80–0.85) and between the change in mid-region VL CSA and isometric force at long muscle lengths (*r* = 0.79–0.95) (Noorkõiv et al., 2014) following isometric training. These results indicate that region-specific changes in CSA may underpin changes in the force-length relationship of muscle, suggesting a functional role of variable hypertrophy. It also appears that the strength of the relationship between the change in isometric torque and region-specific changes in muscle size may be task-dependent, and are stronger following isometric training.

#### Effect of Muscle Size

In the present study, the change in maximum isometric torque was most strongly associated with the change in muscle size (*r* = 0.36 to 0.45, *p* < 0.05, for proximal-region whole quadriceps (Q), and VL CSA, and mid-region VL CSA; Table 6). This finding is similar to other studies examining the influence of dynamic strength training (Erskine et al., 2010). While these data suggest a possible causative link between changes in CSA and changes in isometric torque production, a majority of the variance in torque production following training was left unexplained. Therefore, factors other than muscular hypertrophy must contribute significantly to changes in strength in many individuals, and changes in hypertrophy alone may not always be expected to result in notable changes in strength.

#### Effect of Fascicle Angle

The inclusion of fascicle angle in combination with CSA (both measured proximally) improved the predictive strength of the models, which emphasizes the potential importance of physiological CSA (PCSA) for maximizing changes in strength. Increases in fascicle angle allow more contractile tissue to attach to a given area of tendon or aponeurosis (Otten, 1988; Kawakami et al., 1993; Blazevich et al., 2006; Ikegawa et al., 2008) and should thus increase PCSA and, therefore, contractile force. An alternative explanation is that an increase in fascicle angle can increase fascicle rotation during contractions, which produces a gearing effect allowing fascicles to work at slower speeds and enhancing muscle force through the optimization of both the force-velocity and the force-length characteristics (Hill, 1938; Blazevich and Sharp, 2005; Brainerd and Azizi, 2005). However, only high-force (slow-speed) contractions were examined in this study so it is probable that fascicle rotation would be minor (Azizi et al., 2008) and this mechanism may not be of substantial influence. Therefore, it is more likely that the increased ability to pack contractile tissue onto the tendon and aponeurosis was the main benefit derived from the simultaneous increases in CSA and fascicle angle in the proximal region. It is not surprising that proximal VL CSA was most strongly correlated with isometric torque as VL is the largest quadriceps component (Narici et al., 1992). Its proximal CSA is slightly larger than its mid-region CSA (Table 2), and proximal CSA was observed to be strongly predictive of strength differences in a cross-sectional analysis (Trezise et al., 2016). It is not yet known if there is a specific functional importance of the proximal quadriceps muscle during knee extension, but the results of the present study suggest that CSA obtained at the proximal region may be more influential than the middle (or distal) region on individual variations in the change in isometric knee extension torque following training. The result emphasizes the need to examine changes in proximal quadriceps musculature rather than obtaining CSA from a single mid-muscle region or measuring whole muscle volume.

When considering single variable correlations rather than the models, it was of interest that the changes in proximal VL fascicle angle were moderately correlated with the change in isometric torque (*r* = 0.41, *p* < 0.05, Table 6) whilst fascicle angles measured at other locations were not significantly correlated. Significant increases in fascicle angle were elicited by the training at all measurement sites (Table 2), however, these changes were highly variable between individuals (see SD in Table 2). Considering the apparent influence of the proximal region on changes in isometric torque (described above), the possibility exists that the functional influence of other regions was minimal. One previous study also examined the relationship between the change in VL fascicle angle and the change in isometric torque following a similar training protocol to that used in the present study, with weak and non-significant correlations reported (*r* = −0.33, *p* = 0.21) (Erskine et al., 2010). Given these results it may be concluded that changes in fascicle angle, when considered in isolation, are relatively unrelated to changes in isometric strength, however, they are possibly important when changes in CSA also occur simultaneously.

#### Effect of Fascicle Length

Changes in fascicle length should, theoretically, be associated with increases in muscle shortening speed and force production during high speed or large range of movement activities (Sacks and Roy, 1982; Abe et al., 2000; Blazevich and Sharp, 2005). Given this, it was not surprising that fascicle length was not included in any of the best-fit models for the prediction of isometric torque. While Erskine et al. (Erskine et al., 2010) reported a weak correlation between VL fascicle length and isometric torque (*r* = −0.47, *p* = 0.06), Noorkoiv et al. (Noorkõiv et al., 2014) found no relationship between the change in VL fascicle length and the change in isometric torque. In the present study, there was also no correlation observed between the change in VL fascicle length and the change in isometric torque (Table 6). The lack of relationships observed between fascicle length and isometric torque indicate that fascicle length change may have little functional influence on isometric torque, at least when measured at the angle of peak torque. In future research, the impact of fascicle length on torque production at long versus short muscle lengths might be more explicitly examined.

### Factors Associated With Changes in Concentric and Eccentric Torque

The best-fit models for predicting the changes in concentric (EMG:M_AVEQ_ + CSA,VL_PROX_ + θ_f_VI) and eccentric (EMG:M_AVEQ_ + CSA,Q_PROX_ + θ_f_VI) torque displayed moderate relationships (*R*^2^ = 0.40 and 0.41, for concentric and eccentric torque, respectively). While the inclusion of changes in CSA and fascicle angle may again indicate the importance of an increase in contractile tissue within the muscles for strength change following training, the change in muscle activation was also included in all candidate models (Table 4) and was also the most strongly correlated with the change in torque of any neuromuscular variable (*r* = 0.51 to 0.56 for the change in a both average quadriceps (EMG:M_AVEQ_) and RF (EMG:M_RF_) muscle activity and the change in concentric and eccentric torque, respectively, Table 6). Muscle activity can, therefore, be considered the most important variable influencing concentric and eccentric torque production in the present study.

#### Effect of Agonist Muscle Activation

Whilst a greater agonist muscle activity is often considered to be an important factor influencing strength expression (Narici et al., 1989; Häkkinen et al., 1992; Aagaard et al., 2000b, 2002; Del Balso and Cafarelli, 2007), the relationship between the change in muscle activity and the change in torque has not been well studied. Researchers have commonly used EMG procedures to assess changes in muscle activity (Narici et al., 1996; Aagaard et al., 2000b), however, peripheral factors can strongly influence these measurements (Farina et al., 2004). To account for the potential influence of peripheral changes on EMG amplitudes in the present study, EMG signals were normalized to their respective M-wave amplitudes (elicited by supramaximal femoral nerve stimulation). M-wave-normalized EMG amplitudes (EMG:M) were considered to provide a clearer estimate of central drive because alterations at, and distal to, the neuromuscular junction, including changes to muscle membrane excitability and fascicle angulation, should be removed by the M-wave normalization process (Fuglevand et al., 1993). In fact, %VA (obtained using the interpolated twitch technique) and quadriceps EMG:M amplitudes measured during the isometric contractions were both found to increase over the training period in the present study, which is some support for the supposition.

Moderate correlations were observed between the change in both concentric and eccentric knee extension torque and the percent change in average quadriceps EMG amplitude (EMG:M_AVEQ_; *r* = 0.52, *p* < 0.01 and *r* = 0.56, *p* < 0.001, respectively; Table 6). Therefore, those individuals who displayed a greater increase in agonist EMG:M amplitude also displayed greater improvements in torque when measured during dynamic contractions. Among the quadriceps components, the percent change in RF EMG amplitude (EMG:M_RF_) was most strongly related to the changes in both concentric (*r* = 0.56, *p* < 0.001) and eccentric (*r* = 0.51, *p* < 0.01) torque, while the percent changes in VM and VL showed either weak or no relationship with the change in torque for either measure (Table 6). These results are similar to Higbie et al. (Higbie et al., 1996) (*r* = 0.48 and 0.68, *p* < 0.05, for eccentric and concentric contractions, respectively), who considered the strength of this correlation reasonable considering EMG is not reflective of all possible neural adaptations following training. Thus, models incorporating either the change in average quadriceps or rectus femoris amplitude both had substantial support for predicting the change in both concentric and eccentric torque following training.

Other muscle activity measures (i.e., voluntary activation and antagonist) were collected simultaneously with agonist EMG:M in the present study with the intention of strengthening evidence for the change in muscle activity in the regression models. In fact, the change in %VA was included along with ΔEMG:M_RF_ in the models with strong support for predicting the change in eccentric torque (Table 5). The change in %VA obtained during isometric contraction (at the relevant angle of maximum isometric or eccentric torque) showed no relationship with the change in isometric torque (*r* = 0.23) despite it being shown to be an important predictor of maximum isometric and eccentric torque cross-sectionally (Trezise et al., 2016). This differs somewhat from the results observed by Erskine et al. (Erskine et al., 2010) (*r* = 0.47) in their untrained individuals following 9 weeks of strength training, and also to Stragier et al. (2016) who found a moderate correlation (*r* = 0.73) following 24 weeks of plantarflexor strength training in older adults. In the present study, the difference in correlations between the change in torque and the changes in EMG:M and %VA is understandable, for while %VA is accepted as a good indicator of activation capacity it may be influenced by other factors distal to the neuromuscular junction, including the efficiency of force transmission through the series elastic components (Taylor, 2009), and this may influence the correlations (Table 6). It was also measured during isometric contractions, and thus measurement obtained during dynamic contractions in future studies may yield different results. Regardless, as the eccentric models in which both EMG:M and %VA were included received substantial support (ΔAIC_c_ < 2) it is probably the case that both measurements provide unique (i.e., different) information with regard to muscular force production.

#### Effects of Antagonist Muscle Activation

Antagonist muscle activity may also influence maximal torque production by decreasing net joint torque (Narici et al., 1996; Kellis and Baltzopoulos, 1998; Aagaard et al., 2000a); however, no changes in biceps femoris EMG amplitudes (EMG_BF_) were observed after training in any contraction condition, which is consistent with the findings of Reeves et al. (Reeves et al., 2004). The large inter-individual variability in this change (see large SD; Table 1) should have made relationships more, rather than less, likely to be detected, however, despite being considered to influence maximum torque production (Trezise et al., 2016), no relationships were observed between the changes in torques and the change in antagonist activity. Therefore, changes in other functional and structural variables were more clearly associated with changes in dynamic torque production.

#### Effect of Fascicle Angle

Based on the influence of fascicle angle on isometric, concentric and eccentric torque, it might be speculated that changes in fascicle angle, when considered in isolation, are relatively unimportant for strength increases (in the present study, only VI fascicle angle was correlated with the change in concentric torque; Table 6). However, the present results show that the inclusion of fascicle angle simultaneous with CSA or muscle activity variables substantially increased model strength. The specific importance of VI fascicle angle in the models cannot be readily explained, especially given that VL fascicle angle appeared more important for the change in isometric torque production. Speculatively, VI may play a more functional role during dynamic contractions (i) than other quadriceps components and (ii) than in isometric contractions, as it is a large muscle that anchors proximally to a bony attachment at its endpoint [VL contacts the aponeurotic sheath of VI; (Ando et al., 2018)]. VI muscle activity was not measured in the present study and thus this hypothesis cannot be examined herein, although it may be done in the future (Akima and Saito, 2013; Watanabe and Akima, 2011). At his point, however, there is no information regarding potential improvements in VI activity following strength training. As the change in VI fascicle angle was smaller than in other quadriceps components, and also displayed a relatively small range of change (Table 2), statistical effects are unlikely to underpin its inclusions and this may indicate a particular functional importance. Speculatively, VI may have a greater influence on strength change when increases in muscle size and/or activation [or other changes that could not be examined in the present study, such as lateral force transmission efficiency (Huijing, 2003)] occur. Thus, while not predictive in isolation, the simultaneous changes in proximal quadriceps CSA, VI fascicle angle and quadriceps muscle activity appear to strongly influence the change in concentric and eccentric torque production following strength training.

#### Effect of Fascicle Length

Models incorporating the change in VL fascicle length measured mid-muscle received substantial support for inclusion in the concentric models. In isolation, this measure was also weakly correlated with the change in concentric torque (*r* = −0.34, *p* < 0.05), indicating that individuals who demonstrated the least increase (or a decrease) in fascicle length showed a greater increase in torque after training. Of interest, RF fascicle length changes were the most strongly correlated with the change in eccentric torque of all the muscular variables (*r* = −0.43, *p* < 0.05). Thus, while fascicle length is often considered to be associated with force production during higher-speed (lower-load) movements, the current relationships indicate that changes in fascicle length may have some influence on the change in slower-speed (higher-load) dynamic strength following heavy strength training.

#### Effect of (Pre-training) Moment Arm

A large moment arm is theoretically ideal for high torque production, whereas a small moment arm optimizes joint angular range and velocity (Lieber and Fridén, 2001; Smith et al., 2007), and moment arm distance may influence the magnitude of strength improvement after a period of strength training. In the present study, models incorporating moment arm distance also received substantial support (ΔAIC_c_ < 2) for inclusion in the eccentric models, and moderate support for consideration in both the isometric and concentric (data not shown) models (ΔAIC_c_ < 4). Therefore, while not incorporated within the best-fit models, moment arm distance does appear to influence the change in joint torque, with greater improvements in dynamic torque production observed in individuals with a greater moment arm distance.

### Are Variables Associated With Strength in a Cross-Sectional Analysis Also Influential to Longitudinal Strength Change?

In our previous study (Trezise et al., 2016), several neuromuscular variables were identified as being strongly correlated with maximum isometric and concentric torque production in a group (*n* = 56) of healthy young men. Those results suggested that the targeting of these variables might allow for increases in muscular strength. However, the best-fit models identified in that study did not predict the changes in strength measured in this study (*R*^2^ ≤ 0.07; Table 5). For example, muscle activation was strongly related to maximum isometric torque cross-sectionally, but its change was not a strong predictor of the change in isometric torque following training. Similarly, joint moment arm distance was an important predictor of maximum concentric torque cross-sectionally, but was not found to be a predictor of the change in concentric torque with training. This clearly indicates that the results of cross-sectional and longitudinal studies may differ substantially and conclusions must be made specifically to the study design used. Thus, the functional importance of specific anatomical and neuromuscular variables for muscular strength appears to be contextual, for while strength variation within a population may be well explained by variations in muscle size, activity, architecture and moment arm, the changes in strength elicited by strength training of the duration used in the present study cannot be clearly linked with changes in these specific neuromuscular variables. Longer training periods eliciting greater strength changes may be required before clearer indications can be seen, or factors not measured in the present study [e.g., lateral force transfer; (Huijing, 2003)] might be influential with regards to strength change. Further research using longitudinal designs is required in order to provide the information necessary to allow for the specific targeting of neuromuscular factors that most clearly influence strength change.

Another factor influencing the strength of the relationship between the change in torque and the change in muscle size (and, in fact, the change in any of the neuromuscular variables) is the magnitude of change elicited by the training, which was far less than the inter-individual variation in these variables within a population. As an example, the ranges of isometric torque and CSA_PROX_ measured at pre-training were 297.1 N⋅m and 63.4 cm^2^, respectively, whilst the ranges of the changes in these variables were only 92.6 N⋅m and 8.8 cm^2^ after training (i.e., 31 and 14% of the variation measured pre-training). Thus, statistically, the variables that demonstrate a greater range of change may display stronger associations with the change in strength following training. This also ensures that the chance of observing strong relationships (*R*^2^ > 0.50) between the prediction models and the change in torque is substantially reduced.

## Summary

In the present study, models incorporating the changes in anatomical and neuromuscular variables explained up to 41% of the variance in the change in torque following 10 weeks (20 sessions) of heavy strength training. These data clearly show the benefit of a modeling approach, since some variables that were poorly correlated in isolation were incorporated into the best models. Thus the interactions between variables is particularly important, and looking at variables in isolation may not lead to correct conclusions. However, even when models were used a large portion of variance remained unexplained. This may be caused by (a) measurement errors, which ensure that the full variance can never be explained by a model, (b) relatively small changes in the magnitude of variables reducing the likelihood of finding strong relationships (i.e., between-subject variability in a population is much larger than within-subject changes with training), and (c) other factors that were not measured (e.g., force transfer efficiency or inter-muscular coordination changes) impacting the changes in strength. Nonetheless, the data might also indicate that the adaptations responsible for strength change vary considerably between individuals, so the effect of any single variable, or combination of variables, on strength change may only be moderate. Hence, the identification of the variables to most strongly target using physical training programs may not be possible at the population level. Instead, identification of anatomical and physiological targets for change might need to be made for each individual prior to the implementation of training programs; a goal of future research is to determine whether these individualized targets can be identified using pre-training testing.

It is noteworthy that the combination of variables found to most influence the change in torque in the present study differed from the combinations that best predicted maximum torque in a cross-sectional analysis (Trezise et al., 2016). This implies that the variables most influencing between-subject variations in muscular strength are not the same as those influencing the within-subject change in strength with training; i.e., results of cross-sectional analyses should not be used to infer likely outcomes of longitudinal analyses. Based on the present data, muscle CSA (especially at the proximal, rather than mid-thigh, region), fascicle angle and muscle activation appear to explain the majority of the inter-individual variances in the change in strength following training due to their incorporation in a majority of the best-fit models. As muscle activation was the strongest predictor of concentric and eccentric knee extensor strength, strength training programs targeting improved muscle activation might generally elicit the greatest improvements in concentric and eccentric knee extensor strength early in a strength training program.

The focus of the present study was the strength and neuromuscular adaptations arising from 10 weeks (20 sessions) of heavy lower-limb strength training, as early stage strength change is an important factor influencing the whole to continue and training program, and is important in a rehabilitation context. Additional studies assessing differing loads, volumes, movement speeds and durations will further our understanding of the interactions between all these variables. Additionally, the results of the study are only applicable to young, healthy men. Future studies assessing different sample groups (i.e., strength trained, elderly, or clinical populations) may also identify different neuromuscular adaptations, and hence relationships, between strength change and anatomical and neuromuscular changes.

## Ethics Statement

Prior to performing in the study, all subjects provided written informed consent and the experimental procedure was approved by the Edith Cowan University Human Research Ethics Committee and confirmed to the Declaration of Helsinki.

## Author Contributions

JT and AB designed the research, wrote the manuscript, and approved the final manuscript for publication. JT conducted the data collection and analysis.

## Conflict of Interest Statement

The authors declare that the research was conducted in the absence of any commercial or financial relationships that could be construed as a potential conflict of interest.
